# Successful Enzyme Colocalization Strategies in Yeast for Increased Synthesis of Non-native Products

**DOI:** 10.3389/fbioe.2021.606795

**Published:** 2021-02-09

**Authors:** Hannah C. Yocum, Anhuy Pham, Nancy A. Da Silva

**Affiliations:** Department of Chemical and Biomolecular Engineering, University of California, Irvine, CA, United States

**Keywords:** enzyme colocalization, organelle targeting, enzyme scaffolds, yeast, *Saccharomyces cerevisiae*

## Abstract

Yeast cell factories, particularly *Saccharomyces cerevisiae*, have proven valuable for the synthesis of non-native compounds, ranging from commodity chemicals to complex natural products. One significant challenge has been ensuring sufficient carbon flux to the desired product. Traditionally, this has been addressed by strategies involving “pushing” and “pulling” the carbon flux toward the products by overexpression while “blocking” competing pathways via downregulation or gene deletion. Colocalization of enzymes is an alternate and complementary metabolic engineering strategy to control flux and increase pathway efficiency toward the synthesis of non-native products. Spatially controlling the pathway enzymes of interest, and thus positioning them in close proximity, increases the likelihood of reaction along that pathway. This mini-review focuses on the recent developments and applications of colocalization strategies, including enzyme scaffolding, construction of synthetic organelles, and organelle targeting, in both *S. cerevisiae* and non-conventional yeast hosts. Challenges with these techniques and future directions will also be discussed.

## Introduction

**Figure 1:**
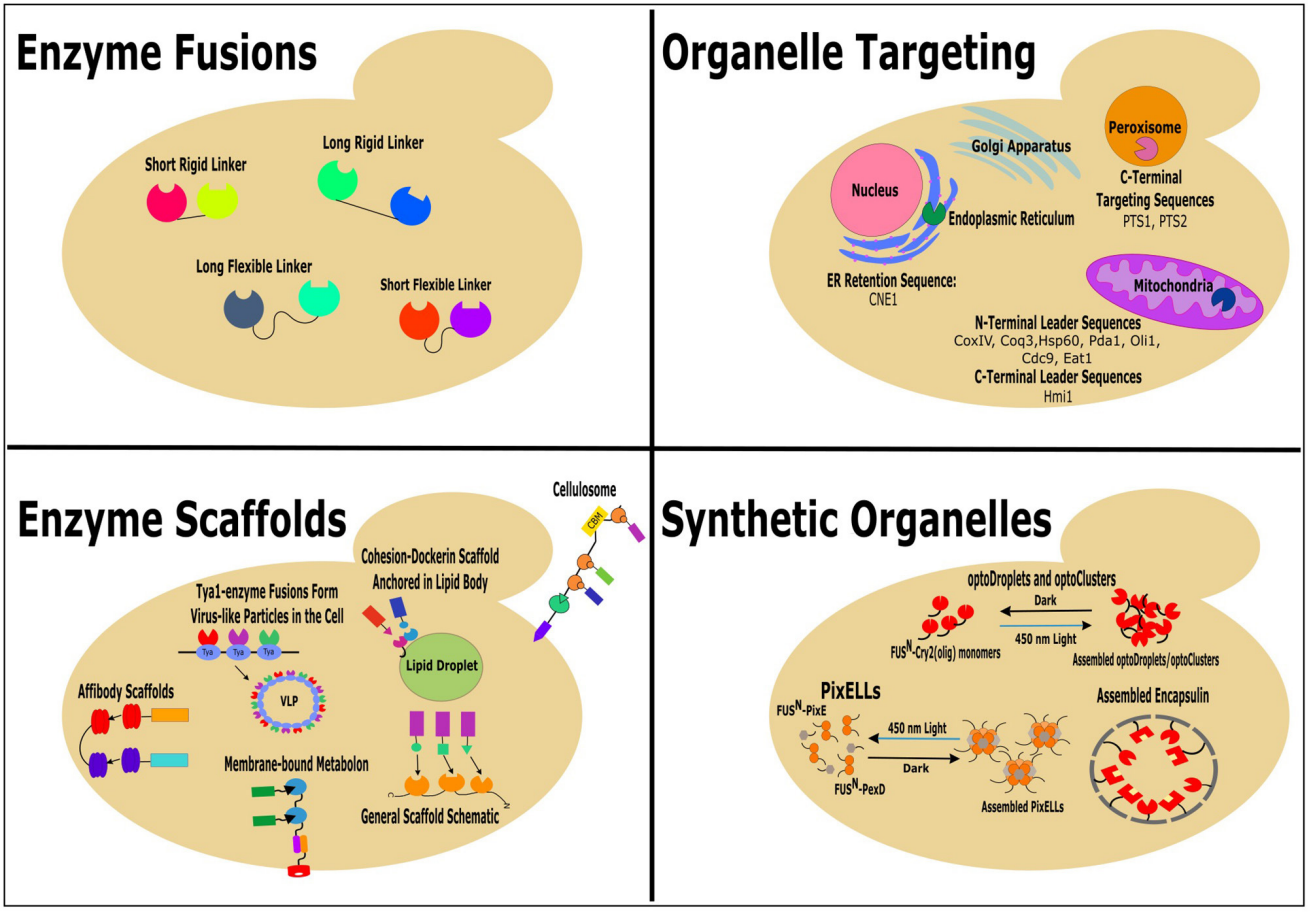
Successful colocalization strategies: enzyme fusions, enzyme scaffolds, organelle targeting, and synthetic organelles, for increasing the production of non-native products in yeast.

A main goal of metabolic engineering is to increase production of non-native products built from native precursors. Traditionally, this has been done by “pushing” or “pulling” the carbon flux toward the product-producing pathway by overexpression of pathway enzymes or by deletion of competing pathways (Ostergaard et al., 2000). Recently, many novel molecular engineering tools and synthetic biology strategies have been successfully employed to improve the production of desired compounds in microbial cell factories (Lian et al., 2018; Liu and Nielsen, 2019; Guirimand et al., 2020; Xu et al., 2020). This review focuses on the latest successful attempts to localize the metabolic pathway enzymes in close proximity for redirection of flux through the pathway of interest and, subsequently, increase synthesis of the products in *Saccharomyces cerevisiae* and other yeast species. The methods include enzyme fusion, enzyme scaffolding, organelle targeting, and construction of synthetic organelles (Figure 1).

## Enzyme Fusions

Construction of synthetic fusion proteins is perhaps the most obvious strategy to enhance the substrate channeling effect in metabolic engineering. By physically fusing successive enzymes in the production pathway together, substrates can be localized in the same vicinity. However, despite the straightforward nature of the approach, fusion proteins are not always successful in improving final product titer, as fusion can lower the enzymatic activities or hinder the folding processes (Jia et al., 2014). Therefore, optimization of the fusion protein construct is often needed.

One critical factor in designing a multi-enzyme complex is the choice of linker. Albertsen et al. (2011) demonstrated the effect of different linkers on the production of patchoulol for a two-enzyme fusion of farnesyl diphosphate synthase (FPPS) and patchoulol synthase (PTS). A short flexible linker (GSG) resulted in the highest titer (9.5 mg/L, representing a 2-fold increase over the free enzyme system) whereas a very long linker (the entire CFP protein sequence) resulted in the lowest titer (one third of that with the flexible linker). This study showed a decrease in patchoulol titer as the length of the linker increased; however, since the optimal linker is often dependent on the specific protein, this finding may not translate to other proteins. A comprehensive review of different linkers and their properties (such as length and structural integrity) can be found in Chen et al. (2013).

Interestingly, the order in which the enzymes are fused may also affect the performance of the fusion protein complex. By fusing *Petroselinum crispum* coumarate-CoA ligase (Pc4CL) and *Rheum palmatum* benzalacetone synthase (RpBAS) using either a flexible (VDGGSGR) or rigid (VDEAAAKSGR) linker, Lee et al. (2016) achieved a 6.5-fold improvement in raspberry ketone titer relative to the free enzymes. However, this improvement was only observed with the orientation Pc4CL-RpBAS and not with RpBAS-Pc4CL; for the latter, the final titer was similar to that obtained when expressing free enzymes. Similar findings were also reported in Hu et al. (2017) for the synthesis of germacrene A when fusing the farnesyl diphosphate synthase (*ERG20*) and a *Lactuca sativa* germacrene A synthase (*LTC2*_*op*__t_) with two different flexible linkers, GSG and GGGGS. No significant differences were observed for the linkers used and both protein orientations resulted in higher germacrene A production relative to free enzymes. However, the *ERG20-LTC2*_*opt*_ configuration resulted in 97.7 mg/L germacrene A production compared to approximately 60 mg/L for the *LTC2*_*opt*_*-ERG20* configuration.

Recently, Rabeharindranto et al. (2019) reconfirmed the importance of both linkers and domain order when constructing a synthetic fusion protein for carotenoid synthesis. A tri-domain enzyme containing the full β-carotene synthesis pathway (CrtY, CrtB, and CrtI) was constructed using different domain orders and linkers. The best tri-domain enzyme [CrtY linked with CrtB by their natural 27 residue linkers and CrtB linked with CrtI by an (EAAAK)_4_ linker] resulted in a 2-fold increase in β-carotene production relative to the native system. Another successful three-enzyme fusion protein in *S. cerevisiae* was a trifunctional cellulase complex, with each enzyme connected by the flexible GGGGS_3_ linker (Liu et al., 2018). This fusion protein had a 46, 6.7, and 46% increase in β-glucosidase, exoglucanase, and endoglucanase activity, respectively, when compared to individual free enzymes.

## Enzyme Scaffolds

Protein scaffolds, where specific enzymes are recruited onto synthetic constructs, have been used to localize desired biosynthetic pathway enzymes to improve product formation via substrate channeling. Dueber et al. (2009) successfully demonstrated the use of synthetic scaffolds comprised of metazoan protein-protein interaction domains and ligands in *Escherichia coli*, increasing mevalonate production 77-fold. There were concerns about the efficiency of this approach for *S. cerevisiae*, since native proteins may interact with the scaffold domains (Siddiqui et al., 2012). However, the metazoan synthetic scaffold improved the production of resveratrol in *S. cerevisiae* by 5-fold relative to the non-scaffold system and by 2.7-fold relative to a protein fusion strategy (Zhang et al., 2006; Wang and Yu, 2012). Nonetheless, optimization of the system was still important; depending on the stoichiometry of the SH3 and pDZ domains in the protein scaffold, the increase in resveratrol production varied from 1.2 to 5-fold over the control.

Another successful synthetic scaffold in *S. cerevisiae* is based on the cohesin and dockerin-enzyme interaction found in the surface cellulosomes of *Clostridium cellulovorans* and other microorganisms (Mechaly et al., 2001). Engineered cellusome-like complexes localizing cellulases have been successfully expressed on the surface of *S. cerevisiae* to improve ethanol production (Fan et al., 2012; Tsai et al., 2013; Tang et al., 2018). Recently, the “largest cellulolytic complex” was successfully constructed on the surface of the yeast *Kluyveromyces marxianus* (Anandharaj et al., 2020). However, utilization of the dockerin-cohesin interaction as an intracellular metabolic scaffold in yeast has been limited. Kim and Hahn (2014) took advantage of the high binding affinity between cohesion and dockerin (Kd <10^−11^ M) (Stahl et al., 2012) to create a substrate channeling module in the cytosol of *S. cerevisiae*. Increasing numbers (2, 3, or 7) of cohesin domains were included on the scaffold and the C terminal dockerin fusion proteins of heterologous AlsS, AlsD, and endogenous Bdh1 proteins were overexpressed. 2,3-Butanediol production increased by 37% compared to the scaffold-free system in fed-batch fermentation with occasional glucose feeding; although the increase was limited, this was the first study to utilize the interaction between cohesin and dockerin in the yeast cytosol and for metabolic engineering. In a follow-up study (Kim et al., 2016), this synthetic substrate channeling strategy was used to redirect carbon flux from pyruvate to 2,3-butanediol using a heterologous AlsS or to lactate using heterologous LdhA, rather than to ethanol. The native Pyk1 (for pyruvate synthesis) was fused to a cohesin domain and AlsS to a dockerin domain colocalizing the two enzymes, resulting in a 38% increase in 2,3-butanediol production and a 46% decrease in ethanol production compared to the native strain. However, this strategy was unsuccessful in redirecting the flux toward lactate formation; the dockerin-fused LdhA had a >2-fold lower specific activity relative to wildtype LdhA (11.6 and 27.9 U/nmol, respectively). As discussed in the previous section, the reduction of enzymatic activity presents a significant challenge to fusion-based colocalization strategies.

The dockerin-cohesin interaction was also utilized to colocalize the ethyl acetate biosynthesis enzymes onto the surface of lipid droplets (thus combining both scaffolding and organelle targeting strategies), resulting in 2-fold increase in the production rate (Lin et al., 2017). In *S. cerevisiae*, ethyl acetate is synthesized by the enzymes Ald6, Acs1, and Atf1, with only Atf1 targeted to the lipid droplets. To target Ald6 and Acs1, the authors screened and identified the protein oleosin as a promising candidate to direct enzymes of interest to the lipid droplets. Cohesin domains were fused to the oleosin proteins while the corresponding dockerin domains were fused to Ald6 and Acs1. After an extensive screening of promoters and scaffold optimization, the ethyl acetate specific titer increased 1.7-fold compared to the strain without scaffold.

An alternate scaffolding strategy is based on the interactions between affibodies and anti-idiotypic affibodies. The 58-residues affibodies (or Z-domains) are a class of non-immunoglobulin affinity proteins derived from *Staphylococcus aureus* protein A (Löfblom et al., 2010). They possess high specificity and binding affinity toward their target proteins (0.3 pM−10 μM) (Ståhl et al., 2017). In a recent study, Tippmann et al. (2017), utilized the Z_Taq_:anti-Z_Taq_ (Kd = 0.7 μM) and Z_IgA_:anti-Z_IgA_ (Kd = 0.9 μM) interactions to create a functional synthetic scaffold in *S. cerevisiae*. A scaffold linked anti-Z_Taq_ and anti-Z_IgA_, and Z_IgA_ and Z_Taq_ were fused with farnesyl diphosphate synthase and farnesene synthase, respectively. By optimizing the amino acid linkers and the enzyme:scaffold ratio, a 135% increase in the yield of farnesene on glucose was achieved. This affibody scaffold was also functional in *E. coli*, in which its utilization resulted in a 7-fold increase in PHB production, demonstrating its versatility as a scaffold platform for metabolic engineering.

Scaffolds were also used to create an artificial Gal2-xylose isomerase complex to improve xylose utilization in *S. cerevisiae* (Thomik et al., 2017). In this study, WASP-homology 1 (WH1) from rat N-WASP was used as the scaffold construct and a xylose isomerase (XI) was fused with WH1 ligand (WH1L) at the N-terminus. To recruit the XI-WH1 scaffold complex to the Gal2 transmembrane protein, a pair of synthetic coiled-coil zippers with high binding affinity for one another was used: SYNZIP1 (SZ1) and SYNZIP2 (SZ2) (Reinke et al., 2010). In this configuration, Gal2 was fused with SZ2 at the N-terminus by either the flexible or helical linker, while the WH1 scaffold was used with SZ1 at the N-terminus by the flexible linker. The synthetic scaffold allowed the Gal2-xylose isomerase complex to form, enabled the yeast strain to uptake and process xylose, and resulted in higher ethanol/xylitol ratio, a proxy measurement for xylose consumption. Kang et al. (2019) used an alternate means to localize pathway enzymes into a multienzyme complex via RIDD and RIAD, short peptides with high binding affinity (K_D_ = 1.2 nM), to improve lycopene production in *S. cerevisiae*. The two key enzymes Idi and CrtE, were tagged with RIAD and RIDD, respectively. The resulting complex yielded 2.3 g/L of lycopene after 144 h fermentation, 58% higher than the control strains.

In a recent study, Han et al. (2018) utilized Tya, a part of the Ty1 retrotransposon, to spatially recruit the key enzymes to improve farnesene and farnesol production in *S. cerevisiae*. Tya is a 49-kDa protein that self-assembles into a shell, similar to virus-like particles (VLPs) (Marchenko et al., 2003). Tya was fused to either the C-terminus or the N-terminus of three key isoprenoid enzymes: tHMG1 (a truncated form of HMG-CoA reductase from *S. cerevisiae*), IspA (from *E. coli*), and AFS1 (from *Malus domestica*) or DPP1 (from *S. cerevisiae*) to create synthetic metabolons to drive carbon flux toward farnesene or farnesol. Titer reached 930 ± 40 mg/L in the best-performing strain, a 3.1-fold increase relative to 300 ± 11 mg/L for free enzymes. The best-performing strain for farnesol production produced 882 ± 15 mg/L, 3.8-fold higher than the control strains expressing free enzymes (231 ± 14 mg/L).

With increased interest in scaffolds to channel substrates toward formation of desired products, additional protein interactions have been screened for their scaffolding potential. Curvature Thylakoid1A (CURT1A) protein, isolated from the thylakoid membrane of *Arabidopsis thaliana*, has been identified as a prospective membrane-bound scaffolding module (Behrendorff et al., 2019). CURT1A can form homo-oligomers in the membrane. By fusing fluorescence proteins to several variants of CURT1A, CURT1A fusion proteins were shown to scaffold onto the endoplasmic reticulum of *S. cerevisiae*, resulting in the fluorescence signal being localized to the membrane.

## Organelle Targeting

Another strategy to colocalize enzymes is to target the enzyme pathway to an organelle. This is done by fusing specific leader sequences to the enzymes, allowing the transporters of the respective organelles to recognize and facilitate entry of the enzymes. Once in the organelles, the semipermeable membrane prevents enzymes from diffusing out to the cytosol and localizes them in a smaller volume, increasing the chances of reaction along the enzymatic pathway. Furthermore, some organelles have different local conditions than the cytosol and those conditions may be advantageous to product formation for a given pathway. The mitochondria and the peroxisomes are the most popular organelles for pathway targeting for the production of non-native products, though the endoplasmic reticulum has also been utilized (Hammer and Avalos, 2017).

### Mitochondria

The mitochondrial matrix is enclosed by two membranes and has a higher reducing redox potential, higher pH, and lower oxygen concentration than the cytosol (Hu et al., 2008; Orij et al., 2009). The mitochondrion is the cellular location for heme and iron-sulfur cluster biosynthesis (Mühlenhoff and Lill, 2000), formation of several amino acids, and the TCA cycle, and also houses a large number of cofactors and metabolites [NADP(H), NADP+, acetyl-CoA, FAD, TCA cycle intermediates] (Malina et al., 2018). In particular, mitochondria are an attractive location for pathways utilizing acetyl-CoA as the acetyl-CoA pool is 20–30 times greater than the cytosolic pool in *S. cerevisiae* (Wagner and Alper, 2016). A mitochondrial leader sequence (MLS) is required for an enzyme to enter the mitochondria, with the CoxIV MLS as the most commonly used (Hurt et al., 1985; Farhi et al., 2011; Avalos et al., 2013). In addition, several other leader sequences, Hmil (Lee et al., 1999), Coq3 (Yuan and Ching, 2016), Hsp60, Pda1, Oli1 (Ehrenworth et al., 2017), Cdc9 (Willer et al., 1999), and Eat1 (Löbs et al., 2018), have been characterized for targeting of non-native enzymes.

Exploiting the high acetyl-CoA pool in the mitochondria has allowed for increased production of terpenes and sesquiterpenes, products that come directly from acetyl-CoA. Introduction of the farnesyl pyrophosphate biosynthetic pathway to the mitochondria resulted in production of 427 mg/l of amorpha-4,11-diene and provided evidence that the mitochondrial membranes prevented diffusion of intermediates to competing cytosolic pathways (Yuan and Ching, 2016). Similarly, targeting the entire mevalonate pathway along with the genes *FPS* and *GES* resulted in a 6-fold improvement in geraniol production; when coupled with cytosolically expressed *G8H, GOR*, and *ISY*, the compounds 8-hydroxygeraniol and nepetalactol were produced in yeast for the first time (Yee et al., 2019). Interestingly, targeting part of the isoprene pathway to the mitochondria resulted in a 2.1 or 1.6-fold increase in isoprene production relative to purely mitochondrial or cytosolic expression, respectively (Lv et al., 2016), highlighting the importance of local environment on enzyme expression.

In addition to acetyl-CoA, there are many other substrates present in the mitochondria in high concentration that can be utilized. Farhi et al. (2011) took advantage of the high concentration of farnesyl diphosphate (FDP) in the mitochondria to increase production of valencene and amorphadiene by 8- and 20-fold, respectively over a cytosolic expression strain. Targeting the entire isobutanol pathway to the mitochondria and utilizing mitochondrial pyruvate increased isobutanol production by 2.6-fold over cytosolic expression (Avalos et al., 2013). Additionally, targeting the 2-ketoacid elongation enzymes Leu4, Leu1, and Leu2 (responsible for the cyclic elongation of iso-alcohol products by one carbon per cycle) to the mitochondria increased isopentanol production over isobutanol by 53% relative to cytosolic production (Hammer et al., 2020). In the non-conventional yeast *Kluyveromyces marxianus*, targeting of the isobutanol pathway to the mitochondria resulted in 1.1 g/l isobutanol in 250 ml shake flasks compared to 0.03 g/l for the cytosolic pathway, and a titer of 21.6 g/l isobutanol for the mitochondrial-targeted pathway in fed-batch reactors (Patent Application: Buelter et al., 2010).

### Peroxisomes

The yeast peroxisome is the site of β-oxidation as well as the organelle responsible for the removal and degradation of hydrogen peroxide (Van Der Klei and Veenhuis, 1997). Peroxisomes are enclosed by a single membrane and are selectively permeable, with all entering and exiting proteins receiving assistance by membrane-bound proteins called porins (Sibirny, 2016). The size and number of peroxisomes present in a cell is also dynamically controlled and varies with growth conditions (Saraya et al., 2010). Since the peroxisome is the site of fatty acid degradation by β-oxidation and acetyl-CoA is a byproduct of β-oxidation (Poirier et al., 2006), this organelle is promising for the targeting of lipidic product pathways and acetyl-CoA utilizing pathways.

Proteins enter the peroxisome by the use of peroxisomal targeting sequences, PTS1, PTS2, and mPTS (Sibirny, 2016). The need for a quick, efficient peroxisomal targeting sequence can be important to prevent reactions in the cytosol prior to peroxisomal compartmentalization. DeLoache et al. (2016) developed an enhanced efficiency PTS1 (ePTS1) by adding basic residues upstream of the PTS1 targeting sequence. This new ePTS1 was found to import proteins quicker than PTS1 and with less interaction with cytosolic pathways. Additionally, ePTS1 can be used to target multiple enzymes to the peroxisome with minimal decrease of import efficiency.

Synthesis of the mevalonate pathway derived cytosolic product squalene was compartmentalized to the peroxisome, leading to titers of 1.3 g/l, a 138-fold improvement over cytosolic production; when simultaneously produced in both the peroxisome and the cytosol, titers were as high as 11 g/l in fed-batch culture (Liu et al., 2020). Additionally, the peroxisome acts as a storage vessel for squalene produced in both the cytosol and the peroxisome, leading to reduced degradation of squalene. Synthesis of fatty acid derived products (fatty alcohols and alkanes) was also increased by targeting heterologous pathway enzymes to the peroxisome. Fatty alcohol titers were more than 2-fold higher and alkane titers up to 2-fold higher, shorter chain lengths were observed, and there was a higher alkane to fatty alcohol ratio when enzymes were targeted to the peroxisome (Zhou et al., 2016). Additionally, the carboxylic acid reductase, MmCAR, has 45% higher activity in the peroxisome over the cytosol, demonstrating that the peroxisome has favorable conditions for this enzyme. The titer of olefins, a heterologous product class synthesized from a P450 fatty acid decarboxylase, increased 40% when this enzyme was targeted to the peroxisome, indicating that the peroxisome is a promising location for fatty acid derived products.

Targeting enzymes for the production of fatty acid-derived compounds to the peroxisome also increased product titers in *Yarrowia lipolytica*. There was an 11-fold increase in fatty acid ethyl ester (FAEE) titers and a 3-fold increase in alkane titers, with shorter chain length alkanes being produced (Xu et al., 2016). Titers of methyl ketones were 6.5-fold higher when the pathway enzymes were targeted to the peroxisome relative to cytosolic expression (Hanko et al., 2018).

### Endoplasmic Reticulum

The endoplasmic reticulum (ER) works in conjunction with the Golgi apparatus to sort, transport, and modify newly synthesized proteins and lipids. The ER lumen has an oxidative environment (Tu and Weissman, 2004) and a pH similar to that of the cytosol (Paroutis et al., 2004) making it a possible advantageous location for enzymes requiring an oxidative environment. Targeting to the ER is primarily done by a co-translational mechanism involving an ER recognition signal that facilitates entry to the ER (Young et al., 2001) and additional ER retention sequences are necessary for the enzyme to remain in the ER (Pagny et al., 1999). The ER also has the ability to utilize triacyl glycerides and other neutral lipids (Markgraf et al., 2014) resulting in the presence of acyl-CoA and ACP intermediates, making it a possible location for production of fatty acid-derived compounds from those metabolites (Xu et al., 2016).

Targeting a key enzyme in the opioid production pathway (COR from *Papaver somniferum*) to the ER increased the production of morphine by almost 2-fold while also reducing the amount of the undesirable side product, neomorphine, in *S. cerevisiae* (Thodey et al., 2014). In *Y. lipolytica*, targeting select enzymes in the fatty acid ethyl ester (FAEE) and alkane pathways to the ER led to 19- and 5-fold increases in FAEE and alkane production, respectively (Xu et al., 2016).

## Synthetic Organelles

A new emerging technique to colocalize enzymes in yeast is through the use of synthetic organelles. Synthetic organelles are clusters of enzymes that are encapsulated away from the cytosol, similar to an organelle but not a native structure of the cells. Advantages are similar to those of native organelles (smaller volume, separation from the cytosol, and possible different pH conditions) but with the added advantage of avoiding native organelle processes that may compete or interfere with the actions of the localized enzymes.

Many prokaryotes have native intracellular protein compartments that sequester enzymes from the cytoplasm. An example is the encapsulin nanocompartment native to several different bacteria (Giessen, 2016). Encapsulins are constructed by self-assembly of 60 or 180 identical subunits (20–24 nm or 30–32 nm) depending on the source bacterium. Encapsulins were first used as non-native enzyme sequestering organelles in *E. coli* (Kang et al., 2008; Tanaka et al., 2010; Moon et al., 2014; Giessen, 2016; Nichols et al., 2017), but have since been tested in *S. cerevisiae*. In an initial study, use of the encapsulin nanocompartment from *Myxococcus xanthus* in *S. cerevisiae* resulted in the colocalization of split mVenus, resulting in fluorescence (Lau et al., 2018). This yeast encapsulin can also protect enzymes from native cell proteases.

Another method of constructing synthetic organelles in yeast is via light-inducible enzyme clustering techniques (Zhao et al., 2018). The light-inducible optoCluster and the light-repressible PixELL systems create rigid enzyme clusters that place two or more enzymes in close proximity with little to no diffusion away from the cluster. Both systems were used to direct flux at a branch point in the deoxyviolacein pathway toward deoxyviolacein rather than prodeoxyviolacein (which is formed from the diffuse cytosolic enzymes). These light-inducible synthetic organelles both form and dissociate rapidly, pointing to their possible future use as dynamic synthetic organelles for the production of diverse products (Zhao et al., 2019). Synthetic organelles are thus an emerging technology with potential to influence yeast metabolic engineering.

## Conclusions

Immobilization and colocalization of enzymes are effective ways to increase production of both native and heterologous compounds in yeast. These strategies, including enzyme fusion, scaffolding, and organelle targeting have successfully improved product titers (Table 1) without adversely affecting the cell growth and metabolism. Initial studies with synthetic organelles demonstrated both successful colocalization and redirection of a model pathway, indicating the promise of this emerging technology. Nonetheless, the success of these strategies remains somewhat enzyme-specific and extensive optimization (e.g., enzyme-scaffold ratio) or screening (e.g., linker choice, domain order, and targeting sequence) are needed for successful application. Additional studies should allow more general principles to emerge. It is expected that these colocalization synthetic biology approaches will continue to impact metabolic pathway engineering as more strategies are discovered and optimized.

**Table 1:** Enzyme colocalizations studies in yeast and the resulting improvements in product titer.

**Products**	**Strategy**	**Final titer**	Fold improvement^*^	**Organism**	**References**
Amorpha-4,11-diene	Mitochondria targeting (COX4)	427 mg/L	26.5	*S. cerevisiae*	Yuan and Ching, 2016
Geraniol	Mitochondria targeting (COX4)	43.3 mg/L	6	*S. cerevisiae*	Yee et al., 2019
Isoprene	Mitochondria targeting (COX4)	246 mg/L	1.6	*S. cerevisiae*	Lv et al., 2016
Valencene	Mitochondria targeting (COX4)	1.5 mg/L	8	*S. cerevisiae*	Farhi et al., 2011
Amorphadiene	Mitochondria targeting (COX4)	20 mg/L	20	*S. cerevisiae*	Farhi et al., 2011
Isobutanol	Mitochondria targeting (COX4)	635 mg/L	2.6	*S. cerevisiae*	Avalos et al., 2013
Isopentanol	Mitochondria targeting (COX4)	1.24 g/L	7.3	*S. cerevisiae*	Hammer et al., 2020
Isobutanol	Mitochondria targeting (COX4)	21.6 g/L	36.5	*K. marxianus*	Buelter et al., 2010
Squalene	Peroxisome targeting (ePTS1)	11 g/L	138	*S. cerevisiae*	Liu et al., 2020
Olefins	Peroxisome targeting (PTS1)	0.18 mg/L	1.4	*S. cerevisiae*	Zhou et al., 2016
Alkane	Peroxisome targeting (PTS1)	3.5 mg/L	2	*S. cerevisiae*	Zhou et al., 2016
Fatty alcohol	Peroxisome targeting (PTS1)	193 mg/L	2	*S. cerevisiae*	Zhou et al., 2016
Fatty acid ethyl ester	Peroxisome targeting (SKL)	110.9 mg/L	20	*Y. lipolytica*	Xu et al., 2016
Alkane	Peroxisome targeting (SKL)	11 mg/L	3.4	*Y. lipolytica*	Xu et al., 2016
Methyl Ketone	Peroxisome targeting (SKL)	314.8 mg/L	6.5	*Y. lipolytica*	Hanko et al., 2018
Fatty acid ethyl ester	ER targeting	136.5 mg/L	15	*Y. lipolytica*	Xu et al., 2016
Alkane	ER targeting	16.8 mg/L	5	*Y. lipolytica*	Xu et al., 2016
Morphine	ER Targeting (CNE1)	4.7 mg/L	2	*S. cerevisiae*	Thodey et al., 2014
Patchoulol	Fusion protein (GSG)	9.5 mg/L	2	*S. cerevisiae*	Albertsen et al., 2011
Raspberry ketone	Fusion protein (VDEAAAKSGR)	2.81 mg/L	6.5	*S. cerevisiae*	Lee et al., 2016
Germacrene A	Fusion protein (GGGGS)	190.7 mg/L	3	*S. cerevisiae*	Hu et al., 2017
β-carotene	Tridomain fusion protein	2.7 mg/gDCW	2	*S. cerevisiae*	Rabeharindranto et al., 2019
Resveratrol	Metazoan synthetic scaffold	14.4 mg/L	5	*S. cerevisiae*	Wang and Yu, 2012
2,3-Butanediol	Dockerin-Cohesin scaffold	13.6 g/L	1.4	*S. cerevisiae*	Kim et al., 2016
Ethyl Acetate	Lipid droplet scaffold	15 mg/(L*OD)	1.7	*S. cerevisiae*	Lin et al., 2017
Farnesene	Affibodies scaffold	16 mg/L	1.9	*S. cerevisiae*	Tippmann et al., 2017
Farnesene	Tya scaffold	930 mg/L	3.1	*S. cerevisiae*	Han et al., 2018
Farnesol	Tya scaffold	882 mg/L	3.8	*S. cerevisiae*	Han et al., 2018
Lycopene	Peptide tags	2.3 g/L	1.6	*S. cerevisiae*	Kang et al., 2019

* *Fold improvement reported is due to enzyme colocalization (not other metabolic engineering strategies)*.

## Author Contributions

HY and AP conducted the literature survey. All authors wrote and edited the manuscript.

## Conflict of Interest

The authors declare that the research was conducted in the absence of any commercial or financial relationships that could be construed as a potential conflict of interest.

## References

[B1] Albertsen L. Chen Y. Bach L. S. Rattleff S. Maury J. Brix S.. (2011). Diversion of flux toward sesquiterpene production in *Saccharomyces cerevisiae* by fusion of host and heterologous enzymes. Appl. Environ. Microbiol. 77, 1033–1040. 10.1128/AEM.01361-10 21148687 PMC3028695

[B2] Anandharaj M. Lin Y. Rani R. P. Nadendla E. K. Ho M.-C. Huang C.-C.. (2020). Constructing a yeast to express the largest cellulosome complex on the cell surface. Proc. Natl. Acad. Sci. U.S.A. 117, 2385–2394. 10.1073/pnas.1916529117 31953261 PMC7007581

[B3] Avalos J. L. Fink G. R. Stephanopoulos G. (2013). Compartmentalization of metabolic pathways in yeast mitochondria improves the production of branched-chain alcohols. Nat. Biotechnol. 31, 335–341. 10.1038/nbt.2509 23417095 PMC3659820

[B4] Behrendorff J. B. Y. H. Sandoval-Ibanez O. A. Sharma A. Pribil M. (2019). Membrane-bound protein scaffolding in diverse hosts using thylakoid protein CURT1A. ACS Synth. Biol. 8, 611–620. 10.1021/acssynbio.8b00418 30884945

[B5] Buelter T. Meinhold P. Smith C. Aritiduo A. Dundon C. A. Urano J. (2010). Engineered Microrganisms for the Production of one or more Target Compounds. World Intellectual Property Organization. International Publication Number WO 2010/075504 A2.

[B6] Chen X. Zaro J. L. Shen W.-C. (2013). Fusion protein linkers: property, design and functionality. Adv. Drug Deliv. Rev. 65, 1357–1369. 10.1016/J.ADDR.2012.09.039 23026637 PMC3726540

[B7] DeLoache W. C. Russ Z. N. Dueber J. E. (2016). Towards repurposing the yeast peroxisome for compartmentalizing heterologous metabolic pathways. Nat. Commun. 7:11152. 10.1038/ncomms11152 27025684 PMC5476825

[B8] Dueber J. E. Wu G. C. Malmirchegini G. R. Moon T. S. Petzold C. J. Ullal A. V.. (2009). Synthetic protein scaffolds provide modular control over metabolic flux. Nat. Biotechnol. 27, 753–759. 10.1038/nbt.1557 19648908

[B9] Ehrenworth A. M. Haines M. A. Wong A. Peralta-Yahya P. (2017). Quantifying the efficiency of *Saccharomyces cerevisiae* translocation tags. Biotechnol. Bioeng. 114, 2628–2636. 10.1002/bit.26376 28688209

[B10] Fan L.-H. Zhang Z.-J. Yu X.-Y. Xue Y.-X. Tan T.-W. (2012). Self-surface assembly of cellulosomes with two miniscaffoldins on *Saccharomyces cerevisiae* for cellulosic ethanol production. Proc. Natl. Acad. Sci. U.S.A. 109, 13260–13265. 10.1073/pnas.1209856109 22853950 PMC3421181

[B11] Farhi M. Marhevka E. Masci T. Marcos E. Eyal Y. Ovadis M.. (2011). Harnessing yeast subcellular compartments for the production of plant terpenoids. Metab. Eng. 13, 474–481. 10.1016/j.ymben.2011.05.001 21601648

[B12] Giessen T. W. (2016). Encapsulins: microbial nanocompartments with applications in biomedicine, nanobiotechnology and materials science. Curr. Opin. Chem. Biol. 34, 1–10. 10.1016/j.cbpa.2016.05.013 27232770

[B13] Guirimand G. Kulagina N. Papon N. Hasunuma T. Courdavault V. (2020). Innovative tools and strategies for optimizing yeast cell factories. Trends Biotechnol. 10.1016/J.TIBTECH.2020.08.010. [Epub ahead of print]. 33008642

[B14] Hammer S. K. Avalos J. L. (2017). Harnessing yeast organelles for metabolic engineering. Nat. Chem. Biol. 13, 823–832. 10.1038/nchembio.2429 28853733

[B15] Hammer S. K. Zhang Y. Avalos J. L. (2020). Mitochondrial compartmentalization confers specificity to the 2-ketoacid recursive pathway: increasing isopentanol production in *Saccharomyces cerevisiae*. ACS Synth. Biol. 9, 546–555. 10.1021/acssynbio.9b00420 32049515

[B16] Han J. Y. Song J. M. Seo S. H. Wang C. Lee S.-G. Lee H.. (2018). Ty1-fused protein-body formation for spatial organization of metabolic pathways in *Saccharomyces cerevisiae*. Biotechnol. Bioeng. 115, 694–704. 10.1002/bit.26493 29131321

[B17] Hanko E. K. R. Denby C. M. Sànchez i Nogu,é V. Lin W. Ramirez K. J. Singer C. A.. (2018). Engineering β-oxidation in yarrowia lipolytica for methyl ketone production. Metab. Eng. 48, 52–62. 10.1016/j.ymben.2018.05.018 29852272

[B18] Hu J. Dong L. Outten C. E. (2008). The redox environment in the mitochondrial intermembrane space is maintained separately from the cytosol and matrix. J. Biol. Chem. 283, 29126–29134. 10.1074/jbc.M803028200 18708636 PMC2570890

[B19] Hu Y. Zhou Y. J. Bao J. Huang L. Nielsen J. Krivoruchko A. (2017). Metabolic Engineering of *Saccharomyces cerevisiae* for production of germacrene a, a precursor of beta-elemene. J. Industr. Microbiol. Biotechnol. 44, 1065–1072. 10.1007/s10295-017-1934-z 28547322

[B20] Hurt E. C. Pesold-Hurt B. Suda K. Oppliger W. Schatz G. (1985). The first twelve amino acids (less than half of the pre-sequence) of an imported mitochondrial protein can direct mouse cytosolic dihydrofolate reductase into the yeast mitochondrial matrix. EMBO J. 4, 2061–2068. 10.1002/j.1460-2075.1985.tb03892.x 2998781 PMC554462

[B21] Jia F. Narasimhan B. Mallapragada S. (2014). Materials-based strategies for multi-enzyme immobilization and co-localization: a review. Biotechnol. Bioeng. 111, 209–222. 10.1002/bit.25136 24142707

[B22] Kang S. Lucon J. Varpness Z. B. Liepold L. Uchida M. Willits D.. (2008). Monitoring biomimetic platinum nanocluster formation using mass spectrometry and cluster-dependent H2 production. Angew. Chem. Int. Ed. 47, 7845–7848. 10.1002/anie.200802481 18767197

[B23] Kang W. Ma T. Liu M. Qu J. Liu Z. Zhang H.. (2019). Modular enzyme assembly for enhanced cascade biocatalysis and metabolic flux. Nat. Commun. 10:4248. 10.1038/s41467-019-12247-w 31534134 PMC6751169

[B24] Kim S. Bae S. J. Hahn J. S. (2016). Redirection of pyruvate flux toward desired metabolic pathways through substrate channeling between pyruvate kinase and pyruvate-converting enzymes in *Saccharomyces cerevisiae*. Sci. Rep. 6:24145. 10.1038/srep24145 27052099 PMC4823786

[B25] Kim S. Hahn J. S. (2014). Synthetic scaffold based on a cohesin-dockerin interaction for improved production of 2,3-butanediol in *Saccharomyces cerevisiae*. J. Biotechnol. 192(Part A), 192–96. 10.1016/j.jbiotec.2014.10.015 25456062

[B26] Lau Y. H. Giessen T. W. Altenburg W. J. Silver P. A. (2018). Prokaryotic nanocompartments form synthetic organelles in a eukaryote. Nat. Commun. 9:1311. 10.1038/s41467-018-03768-x 29615617 PMC5882880

[B27] Lee C. M. Sedman J. Neupert W. Stuart R. A. (1999). The DNA helicase, Hmi1p, is transported into mitochondria by a C- terminal cleavable targeting signal. J. Biol. Chem. 274, 20937–20942. 10.1074/jbc.274.30.20937 10409639

[B28] Lee D. Lloyd N. D. R. Pretorius I. S. Borneman A. R. (2016). Heterologous production of raspberry ketone in the wine yeast *Saccharomyces cerevisiae* via pathway engineering and synthetic enzyme fusion. Microb. Cell Fact. 15:49. 10.1186/s12934-016-0446-2 26944880 PMC4779194

[B29] Lian J. Mishra S. Zhao H. (2018). Recent advances in metabolic engineering of *Saccharomyces cerevisiae*: New tools and their applications. Metab. Eng. 50, 85–108. 10.1016/j.ymben.2018.04.011 29702275

[B30] Lin J. L. Jie Z. Ian W. (2017). Synthetic protein scaffolds for biosynthetic pathway colocalization on lipid droplet membranes. ACS Synth. Biol 6, 1534–1544. 10.1021/acssynbio.7b00041 28497697

[B31] Liu G.-S. Li T. Zhou W. Jiang M. Tao X. Liu M.. (2020). The yeast peroxisome: a dynamic storage depot and subcellular factory for squalene overproduction. Metab. Eng. 57, 151–161. 10.1016/j.ymben.2019.11.001 31711816

[B32] Liu Y. Nielsen J. (2019). Recent trends in metabolic engineering of microbial chemical factories. Curr. Opin. Biotechnol. 60, 188–197. 10.1016/J.COPBIO.2019.05.010 31185380

[B33] Liu Z. L. Li H. N. Song H. T. Xiao W. J. Xia W. C. Liu X. P.. (2018). Construction of a trifunctional cellulase and expression in *Saccharomyces cerevisiae* using a fusion protein. BMC Biotechnol. 18:43. 10.1186/s12896-018-0454-x 30005661 PMC6044064

[B34] Löbs A. K. Schwartz C. Thorwall S. Wheeldon I. (2018). Highly multiplexed CRISPRi repression of respiratory functions enhances mitochondrial localized ethyl acetate biosynthesis in kluyveromyces marxianus. ACS Synth. Biol. 7, 2647–2655. 10.1021/acssynbio.8b00331 30354074

[B35] Löfblom J. Feldwisch J. Tolmachev V. Carlsson J. Ståhl S. Frejd T. Y. (2010). Affibody molecules: engineered proteins for therapeutic, diagnostic and biotechnological applications. FEBS Lett. 584, 2670–2680. 10.1016/j.febslet.2010.04.014 20388508

[B36] Lv X. Wang F. Zhou P. Ye L. Xie W. Xu H.. (2016). Dual regulation of cytoplasmic and mitochondrial acetyl-CoA utilization for improved isoprene production in *Saccharomyces cerevisiae*. Nat. Commun 7:12851. 10.1038/ncomms12851 27650330 PMC5036000

[B37] Malina C. Larsson C. Nielsen J. (2018). Yeast mitochondria: an overview of mitochondrial biology and the potential of mitochondrial systems biology. FEMS Yeast Res. 18, 1–17. 10.1093/femsyr/foy040 29788060

[B38] Marchenko A. N. Kozlov D. G. Svirshchevskaya E. V. Viskova N. Y. Benevolensky S. V. (2003). The P1 protein of the yeast transposon Ty1 can be used for the construction of bi-functional virus-like particles. J. Mol. Microbiol. Biotechnol. 5, 97–104. 10.1159/000069980 12736532

[B39] Markgraf D. F. Klemm R. W. Junker M. Hannibal-Bach H. K. Ejsing C. S. Rapoport T. A. (2014). An ER protein functionally couples neutral lipid metabolism on lipid droplets to membrane lipid synthesis in the ER. Cell Rep. 6, 44–55. 10.1016/j.celrep.2013.11.046 24373967 PMC3947819

[B40] Mechaly A. Fierobe H. P. Belaich A. Belaich J. P. Lamed R. Shoham Y.. (2001). Cohesin-dockerin interaction in cellulosome assembly: a single hydroxyl group of a dockerin domain distinguishes between nonrecognition and high affinity recognition. J. Biol. Chem. 276, 9883–9888. 10.1074/jbc.M009237200 11148206

[B41] Moon H. Lee J. Kim H. Heo S. Min J. Kang S. (2014). Genetically engineering encapsulin protein cage nanoparticle as a SCC-7 cell targeting optical nanoprobe. Biomater. Res. 18:21. 10.1186/2055-7124-18-21 26331071 PMC4552281

[B42] Mühlenhoff U. Lill R. (2000). Biogenesis of iron-sulfur proteins in eukaryotes: a novel task of mitochondria that is inherited from bacteria. Bioch. Biophys. Bioenerget. 1459, 370–382. 10.1016/S0005-2728(00)00174-2 11004453

[B43] Nichols R. J. Cassidy-Amstutz C. Chaijarasphong T. Savage D. F. (2017). Encapsulins: molecular biology of the shell. Crit. Rev. Biochem. Mol. Biol. 52, 583–594. 10.1080/10409238.2017.1337709 28635326

[B44] Orij R. Postmus J. Ter Beek A. Brul S. Smits G. J. (2009). *In vivo* measurement of cytosolic and mitochondrial ph using a PH-sensitive gfp derivative in *Saccharomyces cerevisiae* reveals a relation between intracellular PH and growth. Microbiology 155, 268–278. 10.1099/mic.0.022038-0 19118367

[B45] Ostergaard S. Olsson L. Nielsen J. (2000). Metabolic Engineering of *Saccharomyces cerevisiae*. Microbiol. Mol. Biol. Rev. 64, 34–50. 10.1128/mmbr.64.1.34-50.2000 10704473 PMC98985

[B46] Pagny S. Lerouge P. Faye L. Gomord V. (1999). Signals and mechanisms for protein retention in the endoplasmic reticulum. J. Exp. Bot. 50, 157–164. 10.1093/jxb/50.331.157

[B47] Paroutis P. Toure N. Grinstein S. (2004). The PH of the secretory pathway: measurement, determinants, and regulation. Physiology 19, 207–215. 10.1152/physiol.00005.2004 15304635

[B48] Poirier Y. Antonenkov V. D. Glumoff T. Hiltunen J. K. (2006). Peroxisomal β-oxidation-A metabolic pathway with multiple functions. Biochim. Biophys. Acta Mol. Cell Res. 1763, 1413–1426. 10.1016/j.bbamcr.2006.08.034 17028011

[B49] Rabeharindranto H. Castaño-Cerezo S. Lautier T. Garcia-Alles L. F. Treitz C. Tholey A.. (2019). Enzyme-fusion strategies for redirecting and improving carotenoid synthesis in *S. cerevisiae*. Metab. Eng. Commun. 8:e00086. 10.1016/j.mec.2019.e00086 30723675 PMC6350077

[B50] Reinke A. W. Grant R. A. Keating A. E. (2010). A synthetic coiled-coil interactome provides heterospecific modules for molecular engineering. J. Am. Chem. Soc. 132, 6025–6031. 10.1021/ja907617a 20387835 PMC2940225

[B51] Saraya R. Veenhuis M. Van Der Klei I. J. (2010). Peroxisomes as dynamic organelles: peroxisome abundance in yeast. FEBS J. 277, 3279–3288. 10.1111/j.1742-4658.2010.07740.x 20629743

[B52] Sibirny A. A. (2016). Yeast peroxisomes: structure, functions and biotechnological opportunities. FEMS Yeast Res. 16, 1–14. 10.1093/femsyr/fow038 27189367

[B53] Siddiqui M. S. Thodey K. Trenchard I. Smolke C. D. (2012). Advancing secondary metabolite biosynthesis in yeast with synthetic biology tools. FEMS Yeast Res. 18, 21. 10.1111/j.1567-1364.2011.00774.x 22136110

[B54] Ståhl S. Gräslund T. Karlström A. E. Frejd F. Y. Nygren P.-A. Löfblom J. (2017). Affibody molecules in biotechnological and medical applications. Trends Biotechnol. 35, 691–712. 10.1016/J.TIBTECH.2017.04.007 28514998

[B55] Stahl S. W. Nash M. A. Fried D. B. Slutzki M. Barak Y. Bayer E. A.. (2012). Single-Molecule Dissection of the High-Affinity Cohesin-Dockerin Complex. Proc. Natl. Acad. Sci. U.S.A. 109, 20431–20436. 10.1073/pnas.1211929109 23188794 PMC3528535

[B56] Tanaka S. Sawaya M. R. Yeates T. O. (2010). Structure and mechanisms of a protein-based organelle in *Escherichia coli*. Science 327, 81–84. 10.1126/science.1179513 20044574

[B57] Tang H. Wang J. Wang S. Shen Y. Petranovic D. Hou J.. (2018). Efficient Yeast surface-display of novel complex synthetic cellulosomes. Microb. Cell Fact. 17:122. 10.1186/s12934-018-0971-2 30086751 PMC6081942

[B58] Thodey K. Galanie S. Smolke C. D. (2014). A microbial biomanufacturing platform for natural and semisynthetic opioids. Nat. Chem. Biol. 10, 837–844. 10.1038/nchembio.1613 25151135 PMC4167936

[B59] Thomik T. Wittig I. Choe J.-Y. Boles E. Oreb M. (2017). An artificial transport metabolon facilitates improved substrate utilization in yeast. Nat. Chem. Biol. 13, 1158–1163. 10.1038/nchembio.2457 28869594

[B60] Tippmann S. Anfelt J. David F. Rand J. M. Siewers V. Uhlén M.. (2017). Affibody scaffolds improve sesquiterpene production in *Saccharomyces cerevisiae*. ACS Synth. Biol. 6, 19–28. 10.1021/acssynbio.6b00109 27560952

[B61] Tsai S.-L. DaSilva N. A. Chen W. (2013). Functional display of complex cellulosomes on the yeast surface via adaptive assembly. ACS Synth. Biol. 2, 14–21. 10.1021/sb300047u 23656322

[B62] Tu B. P. Weissman J. S. (2004). Oxidative protein folding in eukaryotes: mechanisms and consequences. J. Cell Biol. 164, 341–346. 10.1083/jcb.200311055 14757749 PMC2172237

[B63] Van Der Klei I. J. Veenhuis M. (1997). Yeast peroxisomes: function and biogenesis of a versatile cell organelle. Trends Microb. 5, 502–509. 10.1016/S0966-842X(97)01156-6 9447663

[B64] Wagner J. M. Alper H. S. (2016). Synthetic biology and molecular genetics in non-conventional yeasts: current tools and future advances. Fungal Genet Biol. 89, 126–136. 10.1016/j.fgb.2015.12.001 26701310

[B65] Wang Y. Yu O. (2012). Synthetic scaffolds increased resveratrol biosynthesis in engineered yeast cells. J. Biotechnol. 157, 258–260. 10.1016/j.jbiotec.2011.11.003 22100267

[B66] Willer M. Rainey M. Pullen T. Stirling C. J. (1999). The yeast CDC9 gene encodes both a nuclear and a mitochondrial form of DNA ligase I. Curr. Biol. 9, 1085–1094. 10.1016/S0960-9822(99)80477-1 10531002

[B67] Xu P. Qiao K. Ahn W. S. Stephanopoulos G. (2016). Engineering yarrowia lipolytica as a platform for synthesis of drop-in transportation fuels and oleochemicals. Proc. Natl. Acad. Sci. U.S.A. 113, 10848–10853. 10.1073/pnas.1607295113 27621436 PMC5047176

[B68] Xu W. Klumbys E. Ang E. L. Zhao H. (2020). Emerging molecular biology tools and strategies for engineering natural product biosynthesis. Metab. Eng. Commun. 10:e00108. 10.1016/J.MEC.2019.E00108 32547925 PMC7283510

[B69] Yee D. A. DeNicola A. B. Billingsley J. M. Creso J. G. Subrahmanyam V. Tang Y. (2019). Engineered mitochondrial production of monoterpenes in *Saccharomyces cerevisiae*. Metab. Eng. 55, 76–84. 10.1016/j.ymben.2019.06.004 31226348 PMC6717016

[B70] Young B. P. Craven R. A. Reid P. J. Willer M. Stirling C. J. (2001). Sec63p and Kar2p are recquired for the translocation of SRP-dependent precursors into the yeast endoplasmic reticulum *in vivo*. EMBO J. 20, 262–71. 10.1093/emboj/20.1.262 11226176 PMC140194

[B71] Yuan J. Ching C. B. (2016). Mitochondrial acetyl-CoA utilization pathway for terpenoid productions. Metab. Eng. 38, 303–309. 10.1016/j.ymben.2016.07.008 27471067

[B72] Zhang Y. Li S. Z. Li J. Pan X. Cahoon R. E. Jaworski J. G.. (2006). Using unnatural protein fusions to engineer resveratrol biosynthesis in yeast and mammalian cells. J. Am. Chem. Soc. 128, 13030–13031. 10.1021/ja0622094 17017764

[B73] Zhao E. M. Suek N. Wilson M. Z. Dine E. Pannucci N. L. Gitai Z.. (2019). Light-based control of metabolic flux through assembly of synthetic organelles. Nat. Chem. Biol. 15, 589–597. 10.1038/s41589-019-0284-8 31086330 PMC6755918

[B74] Zhao E. M. Zhang Y. Mehl J. Park H. Lalwani M. A. Toettcher J. E.. (2018). Optogenetic regulation of engineered cellular metabolism for microbial chemical production. Nature 555, 683–687. 10.1038/nature26141 29562237 PMC5876151

[B75] Zhou Y. J. Buijs N. A. Zhu Z. Gómez D. O. Boonsombuti A. Siewers V.. (2016). Harnessing yeast peroxisomes for biosynthesis of fatty-acid-derived biofuels and chemicals with relieved side-pathway competition. J. Am. Chem. Soc. 138, 15368–15377. 10.1021/jacs.6b07394 27753483

